# Prediction of Neoadjuvant Chemoradiotherapy Response in Rectal Cancer Patients Using Harmonized Radiomics of Multcenter ^18^F-FDG-PET Image

**DOI:** 10.3390/cancers15235662

**Published:** 2023-11-30

**Authors:** Hye-Min Ju, Jingyu Yang, Jung-Mi Park, Joon-Ho Choi, Hyejin Song, Byung-Il Kim, Ui-Sup Shin, Sun Mi Moon, Sangsik Cho, Sang-Keun Woo

**Affiliations:** 1 Radiological and Medico-Oncological Sciences, University of Science and Technology, Daejeon 34113, Republic of Korea; hmju@kirams.re.kr; 2 Division of Applied RI, Korea Institute of Radiological and Medical Sciences, Seoul 07812, Republic of Korea; jingue.yang@kirams.re.kr (J.Y.); songhj@kirams.re.kr (H.S.); 3 Department of Nuclear Medicine, Soonchunhyang University Bucheon Hospital, Bucheon 14584, Republic of Korea; nm.jmipark@daum.net (J.-M.P.); 114780@schmc.ac.kr (J.-H.C.); 4 Department of Nuclear Medicine, Korea Institute of Radiological and Medical Sciences, Seoul 07812, Republic of Korea; kimbi@kirams.re.kr; 5 Department of Surgery, Korea Institute of Radiological and Medical Sciences, Seoul 07812, Republic of Korea; uisupshin@kirams.re.kr (U.-S.S.); sms@kirams.re.kr (S.M.M.); whtkdtlr@kirams.re.kr (S.C.)

**Keywords:** harmonized radiomics, machine learning, deep learning, radiochemotherapy, ^18^F-FDG PET

## Abstract

**Simple Summary:**

Neoadjuvant chemotherapy is the standard treatment for locally advanced rectal cancer. Preoperative chemoradiotherapy yields clinically significant tumor regression; while some patients exhibit a minimal response, others exhibit a complete pathologic response. We developed deep learning and machine learning models to predict chemoradiotherapy response across external tests using multicenter data. The machine learning model, which used harmonized image features extracted from ^18^F-FDG PET, showed higher performance and demonstrated reproducibility through external tests compared to the deep learning models using ^18^F-FDG PET images. Our study highlights the feasibility of predicting the chemoradiotherapy response of individual patients using non-invasive and reliable image feature values.

**Abstract:**

We developed machine and deep learning models to predict chemoradiotherapy in rectal cancer using ^18^F-FDG PET images and harmonized image features extracted from ^18^F-FDG PET/CT images. Patients diagnosed with pathologic T-stage III rectal cancer with a tumor size > 2 cm were treated with neoadjuvant chemoradiotherapy. Patients with rectal cancer were divided into an internal dataset (n = 116) and an external dataset obtained from a separate institution (n = 40), which were used in the model. AUC was calculated to select image features associated with radiochemotherapy response. In the external test, the machine-learning signature extracted from ^18^F-FDG PET image features achieved the highest accuracy and AUC value of 0.875 and 0.896. The harmonized first-order radiomics model had a higher efficiency with accuracy and an AUC of 0.771 than the second-order model in the external test. The deep learning model using the balanced dataset showed an accuracy of 0.867 in the internal test but an accuracy of 0.557 in the external test. Deep-learning models using ^18^F-FDG PET images must be harmonized to demonstrate reproducibility with external data. Harmonized ^18^F-FDG PET image features as an element of machine learning could help predict chemoradiotherapy responses in external tests with reproducibility.

## 1. Introduction

More than 100,000 individuals worldwide are diagnosed with rectal cancer annually [1]. Rectal cancer is generally treated with neoadjuvant chemoradiotherapy, and tumor responses to therapy are diverse, with 54–75% of patients experiencing tumor downstaging [2]. The reasons for these changes in treatment response are poorly understood, and there is no exact method for predicting the treatment response [3]. Only 15–27% of patients show no residual viable tumors on pathological examination, pathological complete response (pCR) to chemoradiotherapy, and surgery [4]. An accurate imaging biomarker for predicting and evaluating chemotherapy could the early classification of patients into different prognostic groups and personalized treatment approaches. Early detection of patients who might respond poorly to chemoradiotherapy can provide them the opportunity to undergo surgery and receive enhanced treatments to maximize treatment response.

Medical imaging can be used to noninvasively evaluate therapeutic responses to chemotherapy. Jang et al. developed an MRI-based deep learning model for predicting chemotherapy response in rectal cancer and reported the area under receiver operating characteristic curve (AUC) of 0.76 and an accuracy of 0.85. ^18^F-FDG PET/CT has also been widely used to monitor treatment response in many types of malignancies, stages, and diagnoses. ^18^F-FDG PET can help detect glucose metabolism and reveal tumor characteristics. As the anatomical data obtained from CT in rectal cancer patients can help distinguish between physiological and pathological intestinal absorption [5], ^18^F-FDG PET/CT is generally considered a standard tool for predicting the response to chemotherapy in rectal cancer. The radiomics features of ^18^F-FDG PET/CT can also facilitate the prediction of chemoradiotherapy. Taking this into consideration, researchers are increasingly exploring the potential of incorporating radiomic features from ^18^F-FDG PET/CT scans into predictive models to enhance the accuracy and reliability of forecasting responses to chemoradiotherapy.

Recently, the use of machine learning techniques for large and complex biological data analysis has increased. Deep learning techniques are considered among the most powerful tools and are frequently used in bioinformatics because they can allow the analysis of vast amounts of data. Many radiomics studies utilize features extracted by manual method, and these methods are significantly influenced by the knowledge and experience of individual researchers [6]. Consequently, deep learning techniques for computing task-adaptive feature representations by learning layers of complex features directly from medical images are considered suitable tools for predicting prognosis. Deep learning techniques that can automatically learn representative information from raw image data to decode the radiation expression type of tumors can assist in disease diagnosis, prognostic evaluation, and treatment sensitivity prediction [7]. The model performance of deeper hidden layers for pattern recognition has recently begun to surpass that of classical methods in different fields. One of the most popular deep neural networks is the Convolutional Neural Network (CNN). Random forest (RF) technology, which includes an ensemble of decision trees and naturally integrates feature selection and interaction during learning, is a popular choice in personalized medicine. It is nonparametric, efficient, and has a high predictive accuracy for many types of data. RF model is increasingly being adopted because of its advantages in dealing with small sample sizes, high-dimensional feature spaces, and complex data structures [8].

In oncology research, particularly when assessing rectal cancer responses to therapy, the role of SUVmax and SUVmean values derived from 18F-FDG PET/CT scans has been under critical evaluation, as illustrated by several independent studies. Two independent studies showed that the SUVmax predicted chemotherapy with a specificity and overall accuracy of only 35% and 44%, respectively [9,10]. SUVmean, dissimilarity, and contrast from the neighborhood intensity-difference matrix (NGTDM contrast) were significantly and independently associated with OS [11]. A decrease in metabolic tumor volume (MTV) and total lesion glycolysis (TLG) values was suggested to be an indicator of a positive response to chemotherapy [12]. Chemotherapy response predictions using ^18^F-FDG PET/CT are not sufficiently accurate to distinguish patients showing treatment response from those who respond poorly to the treatment [13]. Several studies have reported that radiation features were scanner or protocol-sensitive, highlighting the importance of harmonizing image features to reduce multicenter variability before pooling data from multiple sites [14,15].

In the present study, we evaluated the use of machine learning to predict chemoradiotherapy responses using radiomics harmonization and demonstrated the reproducibility and repeatability of the findings through rigorous external testing. Our effort is not only to address the limitations of the current methodologies but also to contribute to the development of a more robust and universally applicable predictive model for chemoradiotherapy responses in cancer treatment.

## 2. Materials and Methods

### 2.1. Patient Cohort

**Figure 1:**
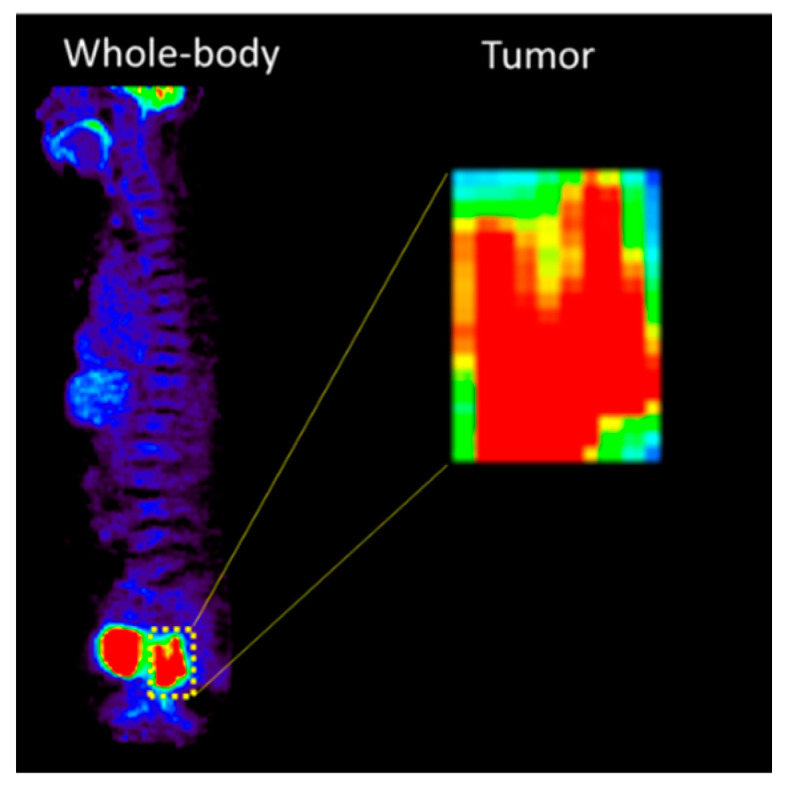
The corp process of rectal cancer region from ^18^F-FDG PET image.

All patients were diagnosed with pathologic T-stage III rectal cancer, with tumor growth into the outer lining of the bowel wall without breaching its integrity. Patients with a tumor size > 2 cm were treated with neoadjuvant chemoradiotherapy before surgery. The internal and external cohorts comprised 116 patients from internal institutions (Korea Institute of Radiological and Medical Sciences) and 40 patients from independent institutions (Soonchunhyang University Bucheon Hospital). The internal cohort comprised 21 patients diagnosed with pCR and 95 patients diagnosed with non-pCR. The external cohort consisted of six patients diagnosed with pCR and 31 patients diagnosed with non-pCR. The rectal cancer region was cropped from an ^18^F-FDG PET image (Figure 1).

### 2.2. Image Feature Extraction

**Figure 2:**
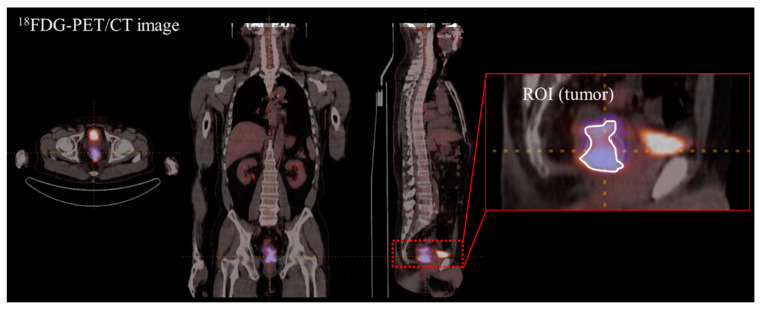
Radiomics extracted from ^18^F-FDG PET/CT.

We utilized LIFEx (Local Image Features Extraction, version 4.90) software to calculate image features from 18F-FDG PET/CT images of rectal cancer patients. In total, 55 image features were extracted. The region of interest (ROI) was marked manually with an SUV threshold of 2.0 (Figure 2). Tumor lesions were identified in the area of ^18^F-FDG uptake, which was pathologically increased and was in contrast to the CT images. To predict chemotherapy response in rectal cancer, first- and second-order images were used separately to compare intensity-based and GLCM-based image characteristics. The AUC was calculated to select the image features from the first- and second-order features using R (version 4.2.2) software (R Foundation for Statistical Computing, Vienna, Austria).

### 2.3. Harmonization Methodology

Harmonization of the image features from the internal and external ^18^F-FDG PET/CT datasets was performed. Both of training set and test set were harmonized in separate manner. The harmonization (ComBat) method was used with an online application (https://forlhac.shinyapps.io/Shiny_ComBat/, accessed on 28 November 2023). ComBat is a batch-matching technology initially proposed for gene expression microarrays [16] and has been widely used in the field of imaging. The ComBat model is given by

*y*^*ij*^ = *α* + *γ*_*i*_ + *δ*_*i*_*ε*_*ij*_

where *j* indicates the specific measurement of image feature *y, i* indicates the setting of the scanner, protocol effect, or even observer effect (called the site effect), α represents the average value of the image features denoted as *y*, *γ_i_* signifies additive batch effect influence on measurement, *δ_i_* represents multiplicative batch effect, and ε*_ij_* is an error term. Batch *i* represents the experimental settings employed for *y* measurement, including the possible scanner effect. Site effects *γ_i_* and *δ_i_* can be estimated using conditional posterior means and subsequently corrected using

$${y_{ij}}^{ComBat}=\frac{y_{ij}-\hat{\alpha }-\hat{\gamma_{i}\;}}{{\hat{\delta }}_{i}}+\hat{\alpha }$$

where $\hat{\alpha },\;\hat{\gamma_{i}\;}$ and ${\hat{\delta }}_{i}$ are estimators of *α, γ_i_* and *δ_i_. y_ij_^ComBat^* is the converted *y_ij_* measured value devoid of the site *i* effect.

### 2.4. Deep Learning and Machine Learning

The CNN structure consisted of input, convolution, batch normalization, ReLU, max pooling, linear, dropout, and output layers. The CNN parameters comprised the optimizer, learning rate, and epoch; the values were set to Adam, 0.0002, and 200, respectively. Two convolutional layers are used. The CNN structure was constructed using two-dimensional input slices taken from each patient. The chemotherapy prediction performance of the RF model was internally and externally evaluated using the scikit-learn library (version 1.2.0) in Python (version 3.10.11).

Augmentation techniques were employed to resolve the data imbalance between pCR and non-pCR. The “RandomRotation” function of PyTorch livery in Python were used to randomly rotate input images by a certain angle to increase the diversity of the training dataset. The “RandomResizedCrop” function of PyTorch livery in Python is employed to randomly select a portion of the input image and subsequently resize it, serving the purpose of augmenting the training dataset and enhancing its variety. The Synthetic minority oversampling technique was implemented on the training dataset for machine learning to mitigate data imbalance.

After splitting the internal dataset at a 7:3 ratio, internal test were performed for both models through evaluating AUC, accuracy, precision, and sensitivity. External test were proceed using independent institution dataset. Confusion matrix-based evaluation metrics including accuracy, sensitivity and precision were estimated and the threshold probability was adjusted to the value that maximizes Youden’s index.

## 3. Results

### 3.1. Patients Cohort

^18^F-FDG PET/CT images from 116 internal and 40 external datasets were used for model estimation. The average ages of the internal and external datasets were 61.85 years and 59.88, respectively. The internal cohort comprised 75 males (64.66%) and 41 females (35.34%). The external cohort comprised 27 males (67.5%) and 13 females (32.5%). A summary of the demographic characteristics and pathological TNM stages is presented in Table 1. The patient cohort included patients who developed lymph node- or distant organ-metastases.

### 3.2. Evaluation of Deep Learning Model

The CNN model for rectal cancer chemoradiotherapy prediction was developed using ^18^F-FDG PET images. The number of pCR data points from the internal and external data increased through augmentation to 84 and 24, respectively. To equalize the amount of pCR and non-pCR data, the pCR data from the internal and external cohorts were decreased by random sampling. The deep learning model showed a performance, with an accuracy of 0.867 and 0.789 in the internal test (Table 2). However, in the external test, the deep learning signature showed an accuracy of 0.557 and 0.355 (Table 3). The deep learning models showed higher performance in internal test then external test.

### 3.3. Image Feature Extraction and Harmonization

**Figure 3:**
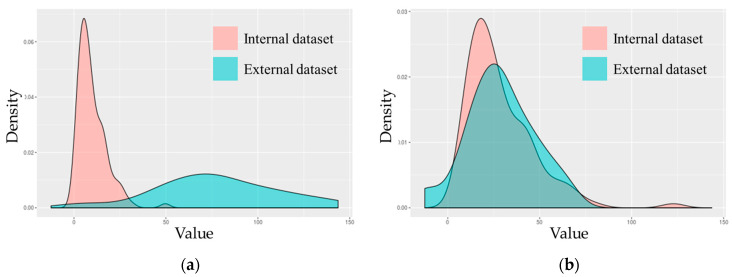
Distribution of GLZLM GLNU value before and after harmonization: (**a**) Distribution of GLZLM GLNU extracted from all T-stage patients before harmonization; (**b**) Distribution of GLZLM GLNU max extracted from all T-stage patients after harmonization.

A total of 55 image featuers were quantitatively calculated from ^18^F-FDG PET and CT images. The image features were separated into first-order features, including conventional indices, shapes, and histogram-based intensity values (n = 23). The image texture features were assigned as second-order features, including a Gray-level co-occurrence matrix (GLCM), neighborhood gray-level difference matrix (NGLDM), Gray-level run-length matrix (GLRLM), and Gray-level zone length matrix (GLZLM) (n = 22) (Figure 2). AUC was calculated to determine image features capable of distinguishing between chemotherapy and non-PCR cases. Subsequently, image features from the internal dataset were selected and used for machine learning. First-order features extracted from ^18^F-FDG PET and CT with AUC over 0.65 and 0.55 were used for machine learning, respectively (Table 4). Second-order features extracted from ^18^F-FDG PET and CT with AUC over 0.7 and 0.6 were used for machine learning, respectively (Table 5). Image feature values from internal and external institutions were harmonized to reduce multicenter variations. GLZLM GLNU, which had the largest change in the distribution of values before and after harmonization, was visualized (Figure 3).

### 3.4. Evaluation of Machine Learning Model

The extracted primary and secondary features were used as variables for the RF model, and each model was evaluated using internal and external tests. The RF model using harmonized first-order features showed an accuracy and AUC of 0.771, which is higher than before harmonization in the external test. The RF model using secondary features exhibited an accuracy and AUC of 0.675 and 0.603 in the external test after harmonization, lower than those without harmonization. The first-order features showed higher accuracy and AUC for the external datasets than the second-order features. In the external test set, the ^18^F-FDG PET image feature as a machine learning signature achieved the highest accuracy with an AUC value of 0.875 and 0.896 (95% confidence interval 0.562–1) (Table 6).

## 4. Discussion

The performance of the machine learning models in predicting chemoradiotherapy response using imaging features extracted from ^18^F-FDG PET images was estimated using an external test. Conducting multicenter studies is one of the main objectives of clinical applications. However, medical images acquired from different institutions may introduce biases due to variations in imaging devices, data acquisition methods, and protocols [17,18]. Because radiomics is sensitive, variations in feature values may occur even in cases where the same feature is extracted from multiple organs. Large-scale radiomic data analysis is required to verify the reproducibility of radiomics, and radiomic features extracted from images acquired from different centers must be integrated. In this study, radiomics harmonization was performed to reduce batch effects. Our results indicated that the harmonization of image features extracted from multiple datasets is essential as a predictor.

In several studies related to cancers, the RF model has shown a high potential in predicting clinical outcomes [19,20,21,22]. The RF model demonstrated reproducibility and repeatability in external tests when utilizing the features extracted from ^18^F-FDG PET images. Because the RF model generates predictions by randomly selecting a decision tree, it mitigates the risk of overfitting. As it traverses the decision tree, it learns the image features that best encapsulate the discriminatory factors for distinguishing tumor characteristics. Moreover, it is expected to yield superior outcomes because it employs an optimal cut-off value for discriminating between pCR and non-pCR patients based on image features. These attributes of the RF model appear to have further enhanced its predictive accuracy and AUC in the context of chemoradiotherapy prognosis.

Medical imaging offers vital insights into the progress of patients with rectal cancer, and AI holds promise for developing quantitative treatment decision support tools. Some studies have shown that tumor metabolic changes on ^18^F-FDG PET were more predictive than tumor morphological modifications on CT [23,24,25]. In our study, image features extracted from ^18^F-FDG PET images showed higher machine learning performance than those extracted from CT images. The imaging features of CT in the external tests showed an accuracy and AUC of 0.425 and 0.593, whereas those extracted from ^18^F-FDG PET showed an accuracy and AUC of 0.875 and 0.896. Our study indicate that the radiomics of ^18^F-FDG PET have a more complementary effect then CT in predicting the pCR of rectal cancer. ^18^F-FDG PET imaging is crucial for monitoring alterations in tumor metabolic activity, playing a vital role in prognostic predictions for patients undergoing concurrent chemoradiotherapy. Although CT imaging provides comprehensive details pertaining to the tumor’s size and shape, excelling in anatomical delineation, it falls short in effectively predicting tumor responses to chemoradiotherapy. This discrepancy highlights a potential limitation in its prognostic utility for this specific therapeutic context. It has been observed that the integration of radiomic features extracted from both ^18^F-FDG PET and CT into predictive models can lead to a decrement in performance, suggesting a paradoxical reduction in the model’s efficacy despite the amalgamation of data from both imaging techniques. This underscores the need for careful consideration when combining features from different modalities to enhance the accuracy of treatment response predictions.

The first and second selected features for AUC values encompassed those previously identified as having prognostic significance in other investigations. The significance of SUVmax, SUVmean, and Uniformity, which are image feature values, has been demonstrated in previous studies. The secondary features based on GLRLM, NGLDM, and GLRM were incorporated as important variables in the radiochemotherapy prediction model. These feature values have demonstrated their predictive utility in various cancers. When the chemoradiotherapy response was predicted using harmonized first-order features, it showed a higher performance than second-order features. The first-order features were derived from histograms, whereas the second-order features were based on the GLCM. As the first-order values exhibited significant alterations following harmonization, the impact of harmonization is noteworthy. Conversely, the second-order values displayed negligible changes after harmonization. Consequently, the model utilizing first-order features exhibited superior performance in predicting rectal cancer chemotherapy outcomes.

There are several ^18^F-FDG PET/CT predictive radiomics for pCR to chemotherapy, including visual response, maximum standardized uptake value (SUVmax), percentage SUVmax reduction, TLG, and MTV [26,27,28,29]. Lovinfosse et al. revealed that SUVmean, dissimilarity, and contrast from contrast NGTDM were significantly and independently associated with OS in patients with rectal cancer. Jean-Emmanuel et al. predicted a complete response using a deep neural network after rectal chemoradiotherapy with 80% accuracy in a multicenter cohort using radiomics extracted from CT. Xiaolu M et al. The RF model for the degree of differentiation, T-stage, and N-stage were obtained using radiomics from MRI (AUC, 0.746; 95% CI, 0.622–0.872; sensitivity, 79.3%; and specificity, 72.2%). Giannini et al. evaluated a logistic regression model using six texture features (five from PET and one from T2w MRI) to determine the chemotherapy outcomes (AUC = 0.86; sensitivity = 86%, and specificity = 83%).

We estimated the performance of the deep learning model in predicting the outcomes of neoadjuvant responses using multicenter ^18^F-FDG PET images. However, the model performance proved insignificant in external tests conducted with datasets from independent institutions. Deep learning demonstrated subpar performance in external tests owing to the omission of dataset harmonization, which failed to account for potential biases between the internal and external datasets. In the case of machine learning, the difference between the internal and external datasets was drastically reduced through the harmonization of the image feature values shown in the ROI; thus, reproducibility as a predictor of machine learning was confirmed. Batch effects can be mitigated by preprocessing the images employed in deep learning, involving techniques such as slope distortion correction, bias slope distortion correction, bias field correction, and intensity normalization, which help standardize the data [30,31]. Reducing batch effects through harmonization at the image level is expected to show high performance in sufficiently predicting chemotherapy, even in external tests.

Our study has some limitations. Deep learning exhibited a lower performance in external tests than in internal tests. This outcome may be attributed to the absence of harmonization between internal and external datasets. Because the CNN model makes predictions using the image itself, it is necessary to harmonize the image. The number of patients within the presently registered external data may be relatively limited, leading to suboptimal performance in external tests. Deep learning techniques in the realm of medical image analysis are challenged by their black-box characteristics, which pose issues for interpretability. Additionally, given the extensive discussion in this article about how chemotherapy and radiotherapy can significantly increase the risk of infertility for women wishing to conceive in the future, we propose a more proactive approach. Women should be given greater autonomy over their reproductive timelines, particularly through the strategic use of oocyte vitrification prior to undergoing such medical interventions [32].

## 5. Conclusions

Our research underscores the critical significance of image harmonization in multicenter studies for accurate chemotherapy response prediction in pancreatic cancer while also highlighting the potential of noninvasive radiomics-based machine learning models in predicting neoadjuvant chemoradiotherapy response in rectal cancer. A machine learning model predicting radiochemotherapy outcomes for pancreatic cancer using harmonized ^18^F-FDG PET imaging features was confirmed to be reproducible and repeatable in external testing using multicenter data. A deep model using ^18^F-FDG PET images without the harmonization process performed poorly in predicting neoadjuvant chemoradiotherapy response, demonstrating the importance of image harmonization in multicenter studies. We confirmed the possibility of using a machine learning model to predict the chemoradiotherapy response of rectal cancer before treatment using radiomics, which can be obtained noninvasively.

## Figures and Tables

**Table 1:** Characteristics of the study cohort.

Characteristics	Internal Dataset (n = 116)	External Dataset (n = 40)
Chemoradiotherapy response (%)		
pCR	21 (18.1)	6 (15)
non-pCR	95 (81.9)	34 (85)
Age (%)		
<65	69 (59.48)	23 (57.5)
≥65	47 (40.52)	17 (42.5)
Mean age (y)	61.85	59.88
Sex (%)		
Male	75 (64.66)	27 (67.5)
Female	41 (35.34)	13 (32.5)
Clinical T-stage, n (%)		
T3	116 (100)	40
Clinical N stage (%)		
N0	19 (16.38)	5 (12.5)
N1	31 (26.72)	8 (20)
N1a	2 (1.72)	
N1b	13 (11.21)	1 (2.5)
N2	37 (31.9)	6 (15)
N2a	13 (11.21)	12 (30)
N2b	1 (0.86)	8 (20)
Clinical M stage (%)		
M0	106 (91.38)	32 (80)
M1	6 (5.17)	
M1a	3 (2.59)	8 (50)
M1b	1 (0.86)	
Clinical stage (%)		
IIA		5 (12.5)
IIB	18 (15.52)	
IIC		
IIIA	42 (36.21)	21 (52.5)
IIIB	46 (39.66)	6 (15)
IIIC		8 (20)
IVA	10 (8.62)	

pCR: pathological complete response.

**Table 2:** Internal test of CNN model using ^18^F-FDG PET images.

	Number of Data		Efficiency Evaluation			
Data Set	pCR	Non-pCR	Accuracy	Precision	Sensitivity	AUC (95% CI)
Imbalanced	21	21	0.867	0.871	0.871	0.903 (0.856–0.949)
Balanced	84	95	0.789	0.843	0.677	0.835 (0.804–0.866)

pCR: pathological complete response; AUC: area under receiver operating characteristic curve; CI: Confidence interval.

**Table 3:** External test of CNN model using ^18^F-FDG PET images.

	Number of Data		Efficiency Evaluation			
Data Set	pCR	Non-pCR	Accuracy	Precision	Sensitivity	AUC (95% CI)
Imbalanced	6	6	0.557	0.542	0.495	0.498 (0.412–0.583)
Balanced	24	25	0.355	0.241	0.475	0.443 (0.378–0.509)

pCR: pathological complete response; AUC: area under receiver operating characteristic curve; CI: Confidence interval.

**Table 4:** Extraction of first-order image features by AUC cut-off value.

First-Order Image Feature			
^18^F-FDG PET	AUC	CT	AUC
SHAPE Sphericity	0.715	Uniformity	0.663
SUVQ1	0.707	Entropy log10	0.659
SUVmean	0.694	Entropy log2	0.659
SUVQ3	0.692	SHAPE Compacity	0.618
SUVQ2	0.69	SHAPE Volume	0.604
Uniformity	0.681	SUVstd	0.6
Entropy log10	0.677	SUVmax	0.593
Entropy log2	0.677	SUVQ3	0.589
SUVstd	0.667	Kurtosis	0.582
SUVmin	0.65	ExcessKurtosis	0.582
		Volume	0.663
		Sphericity	0.579
		Skewness	0.578
		TLG	0.563

Abbreviations: SUVQ, Standardized Uptake Value Quotient; SUV, Standardized Uptake Value; SUVstd, Standardized Uptake Value Standard Deviation; SUVmin, Standardized Uptake Value Minimum; SHAPE, Sphericity, Histogram Analysis, and Parametric Evaluation; SUVmax, Standardized Uptake Value Maximum; TLG, Total Lesion Glycolysis.

**Table 5:** Extraction of second-order image features by AUC cut-off value.

Second-Order Image Feature			
^18^F-FDG PET	AUC	CT	AUC
GLZLM LZLGE	0.766	NGLDM Contrast	0.704
GLZLM LZE	0.765	GLZLM ZP	0.698
GLRLM GLNU	0.763	GLRLM LRE	0.69
GLRLM SRE	0.756	GLRLM RP	0.69
GLRLM RP	0.755	GLRLM SRE	0.689
GLRLM LRE	0.753	GLZLM LZLGE	0.689
NGLDM Contrast	0.74	GLCM Homogeneity	0.689
GLZLM ZP	0.74	GLZLM LZE	0.685
GLZLM LZHGE	0.74	GLZLM LZHGE	0.683
GLCM Homogeneity	0.734	GLCM Energy	0.683
NGLDM Busyness	0.732	GLCM Entropy log10	0.667
GLRLM LRLGE	0.731	GLCM Entropy log2	0.667
GLCM Dissimilarity	0.71	GLCM Dissimilarity	0.661
GLCM Contrast	0.702	GLRLM GLNU	0.647
GLRLM LGRE	0.701	GLRLM LRHGE	0.633
		NGLDM Busyness	0.628
		GLRLM SRHGE	0.617
		GLCM Contrast	0.613
		GLRLM LRLGE	0.613

Abbreviations: GLZLM, Gray-Level Zone Length Matrix; LZLGE, Long Zone Low Gray-level Emphasis; LZE, Low Gray-level Zone Emphasis; GLRLM, Gray-Level Run Length Matrix; SRE, Short Run Emphasis; RP, Run Percentage; LRE, Gray-Level Run Length Matrix; NGLDM, Neighborhood Gray-Level Dependence Matrix; ZP, Zone Percentage; LZHGE, Long-Zone High-Grey level Emphasis; GLCM, Gray-Level Co-occurrence Matrix, LRLGE, Long Run Low Gray-level Emphasis; LGRE, Low Gray-level Run Emphasis.

**Table 6:** Internal and external test of RF model.

Image Feature	Value	Without Harmonization			Without Harmonization			With Harmonization		
		Internal Test			External Test			External Test		
		CT	PET	PET/CT	CT	PET	PET/CT	CT	PET	PET/CT
First order	Accuracy	0.54	0.62	0.56	0.55	0.7	0.525	0.6	0.646	0.771
	Precision	0.524	0.575	0.615	0.227	0.2	0.19	0.222	0.769	0.882
	Sensitivity	0.88	0.92	0.32	0.833	0.333	0.667	0.667	0.417	0.625
	AUC	0.54	0.62	0.56	0.667	0.549	0.583	0.627	0.646	0.771
	95% CI for AUC	-	-	-	0.412–0.921	0.291–0.807	0.325–0.842	0.37–0.885	0.469–0.962	0.429–0.934
Second order	Accuracy	0.52	0.64	0.7	0.425	0.525	0.7	0.65	0.583	0.675
	Precision	0.516	0.63	0.727	0.185	0.19	0.25	0.25	0.7	0.632
	Sensitivity	0.64	0.68	0.64	0.833	0.667	0.5	0.667	0.292	0.5
	AUC	0.52	0.64	0.7	0.593	0.583	0.618	0.657	0.583	0.603
	95% CI for AUC	-	-	-	0.334–0.852	0.325–0.842	0.36–0.876	0.402–0.912	0.562–1	0.344–0.862
All	Accuracy	0.68	0.76	0.7	0.65	0.675	0.775	0.425	0.875	0.725
	Precision	0.765	0.81	0.639	0.214	0.267	0.333	0.185	0.952	0.333
	Sensitivity	0.52	0.68	0.92	0.5	0.667	0.5	0.833	0.833	0.833
	AUC	0.68	0.76	0.7	0.588	0.672	0.662	0.593	0.896	0.77
	95% CI for AUC	-	-	-	0.329–0.847	0.418–0.925	0.556–1	0.334–0.852	0.562–1	0.536–1

AUC: area under receiver operating characteristic curve; CI: Confidence interval.

## Data Availability

The data presented in this study are available on request from the corresponding author. The data are not publicly available due to privacy considerations.
